# The Female Athlete's Heart: Comparison of Cardiac Changes Induced by Different Types of Exercise Training Using 3D Echocardiography

**DOI:** 10.1155/2018/3561962

**Published:** 2018-05-28

**Authors:** Alexandra Doronina, István Ferenc Édes, Adrienn Ujvári, Zoltán Kántor, Bálint Károly Lakatos, Márton Tokodi, Nóra Sydó, Orsolya Kiss, Alexey Abramov, Attila Kovács, Béla Merkely

**Affiliations:** ^1^Semmelweis University Heart and Vascular Center, Városmajor St. 68, H-1122 Budapest, Hungary; ^2^Peoples' Friendship University of Russia, Miklukho-Maklaya St. 6, 117198 Moscow, Russia

## Abstract

We aimed to characterize female athlete's heart in elite competitors in the International Federation of Bodybuilding and Fitness (IFBB) Bikini Fitness category and compare them to athletes of a more dynamic sport discipline and healthy, sedentary volunteers using 3D echocardiography. Fifteen elite female fitness athletes were recruited and compared to 15 elite, age-matched female water polo athletes and 15 age-matched healthy, nontrained controls. Using 3D echocardiography, left ventricular (LV) and right ventricular (RV) end-diastolic volume index (EDVi) and LV mass index (LVMi) were measured. Fitness athletes presented similar LV and RV EDVi compared to healthy, sedentary volunteers. Water polo athletes, however, had higher LV and also RV EDVi (fitness versus water polo versus control; LVEDVi: 76 ± 13 versus 84 ± 8 versus 73 ± 8 ml/m^2^, ANOVA *p* = 0.045; RVEDVi: 61 ± 12 versus 86 ± 14 versus 55 ± 9 ml/m^2^, *p* < 0.0001). LVMi was significantly higher in the athlete groups; the hypertrophy, however, was even more prominent in water polo athletes (78 ± 13 versus 91 ± 10 versus 57 ± 10 g/m^2^, *p* < 0.0001). To the best of our knowledge, this is the first study to characterize female athlete's heart of IFBB Bikini Fitness competitors. The predominantly static exercise regime induced a mild, concentric-type LV hypertrophy, while in water polo athletes higher ventricular volumes and eccentric LV hypertrophy developed.

## 1. Introduction

Athlete's heart is a general term for complex adaptive changes of all four cardiac chambers induced by regular vigorous exercise [1–3]. While in athletes both morphological and functional differences develop compared to nontrained individuals, it has been extensively detailed that the type and intensity of training, age, and gender all significantly influence the cardiovascular alterations [4]. Nevertheless, female athlete's heart is surprisingly underrepresented in current studies despite the importance of gender differences in cardiac adaptation and similar clinical relevance as well.

Traditionally, the various sport disciplines can be divided by their exercise nature to dynamic or static groups [5]. While majority of endurance athletes (dynamic) are presenting eccentric type of left ventricular (LV) hypertrophy, in power athletes (static), however, concentric hypertrophy is more likely to develop [6]. Morganroth's classic hypothesis is barely investigated in context of the female athlete's heart. One of the reasons behind this is that a very low proportion of sport disciplines are characterized by static training predominantly. Nowadays, the unexpected popularity of bodybuilding and fitness makes it possible to investigate female athletes competing in a sport with mainly static exercises and answer the question if Morganroth's division could be applied to women as well.

Accordingly, we aimed to characterize female athlete's heart in elite competitors in International Federation of Bodybuilding and Fitness (IFBB) Bikini Fitness category and compare them to athletes of a more dynamic sport discipline and healthy, sedentary volunteers using 3D echocardiography.

## 2. Methods

Fifteen elite female athletes competing in IFBB Bikini Fitness category were recruited. Furthermore, 15 elite age-matched female water polo athletes (all capped in the national team of the corresponding age category) and 15 age-matched healthy, nontrained (no previous participation in intensive training, <3 hours of exercise/week) women were investigated. Study participants gave prior written informed consent for the examinations (Medical Research Council Approval Number: 077601/2016/OTIG). All of the measurements were performed at least 12 hours after last athletic training of the athletes. Detailed medical history and training regime were obtained along with standard physical examination, blood pressure measurement, and 12-lead ECG. Body composition assessment and conventional and also 3D echocardiography were performed. Subjects with uncommon echocardiographic and/or ECG changes or suboptimal echocardiographic image quality or athletes who suspended regular training in the last 6 months were excluded and did not enter into further statistical analysis.

### 2.1. Body Composition

Weight and height were measured using validated standard equipment. All participants wore light clothing and were barefoot. Body mass index (BMI) was calculated by dividing the body weight by the squared height. Body surface area (BSA) was calculated using the Mosteller formula [7]. Body composition assessment was performed by a Bodystat 1500MDD machine (Bodystat Ltd., Douglas, UK). Participants removed all metal and other objects that could interfere with the scan and were instructed to empty their bladder before the assessment. Each participant was in supine position in the center of the table with palms down and arms beside the body. Age, height, weight, and gender were entered into the machine for performing the automatic calculations. Fat-free mass index (FFMI) was calculated as the fat-free mass (kg), divided by the square of height (m^2^).

### 2.2. Conventional Echocardiography

Echocardiographic examinations were performed on a commercially available ultrasound system (Philips EPIQ 7G, X5-1 transducer, Best, The Netherlands). Standard acquisition protocol consisting of loops from parasternal, apical, and subxiphoid views was used according to current guidelines [8, 9]. In parasternal long-axis view, interventricular septal (IVSd), LV internal (LVIDd), and LV posterior wall (LVPWd) thickness diameters were measured on end-diastolic frame using 2D-guided M-mode technique. Relative wall thickness was calculated by 2xLVPWd/LVIDd. In apical four-chamber view, early (E) and late (A) waves of mitral inflow and deceleration time of E wave were measured using pulsed wave spectral Doppler. Mitral annular lateral, septal, and tricuspid annular systolic (s′), early diastolic (e′), and late diastolic (a′) velocities were measured by pulsed wave Doppler on tissue Doppler imaging [10]. Left atrial (LA) and right atrial (RA) volumes were measured by monoplane Simpson's method and indexed to BSA. In RV-focused apical four-chamber views, basal and mid right ventricular (RV) diameter and RV length were measured. Tricuspid annular plane systolic excursion (TAPSE) was assessed on M-mode recording [11]. Beyond the conventional echocardiographic examination, ECG-gated full-volume 3D datasets reconstructed from 4 or 6 cardiac cycles optimized for the LV or the RV were obtained for further analysis on an offline workstation.

### 2.3. 3D Echocardiography

3D datasets focused on the LV were processed by a single experienced operator using semiautomated, commercially available software (4D LV-Analysis 3, TomTec Imaging GmbH, Unterschleissheim, Germany). We determined end-diastolic (EDVi), end-systolic (ESVi), stroke volumes (SVi), and mass (LVMi) indices. Parameters were normalized to BSA. To characterize LV function, ejection fraction (EF) and deformation parameters such as global longitudinal (GLS) and circumferential strain (GCS) were also assessed.

Offline analysis of the datasets focused on the RV were performed by the same operator using commercially available software (4D RV-Function 2, TomTec). The algorithm automatically generates RV endocardial contour which was manually corrected on multiple short- and long-axis planes throughout the entire cardiac cycle. We quantified RV EDVi, ESVi, and SVi normalized to BSA and EF. Furthermore, the software automatically measures fractional area change (FAC) and free wall longitudinal strain derived from the 3D dataset.

### 2.4. Statistical Analysis

Statistical analysis was performed using dedicated software (StatSoft STATISTICA v12, Tulsa, OK, USA). Data are presented as mean ± SD. Shapiro-Wilk test was used to test normal distribution. One-way ANOVA followed by Fisher post hoc test was used to compare groups, and Pearson or Spearman test was performed for correlation analysis as appropriate. *p* values < 0.05 were considered significant.

## 3. Results

Basic characteristics of the study groups are presented in Table 1. The athlete groups and the healthy, sedentary volunteers were age-matched. Fitness athletes started professional activity for an average of 3.4 ± 1.6 years and trained 12 ± 2 hours a week. Water polo athletes started their career for 12.1 ± 4.6 years and trained 24 ± 8 hours a week. Water polo athletes had higher height, weight, and, correspondingly, BSA. BMI was similar among groups. FFMI was higher in water polo athletes compared to controls and even higher in fitness athletes compared to both the other groups. Systolic blood pressure of water polo athletes was higher. Heart rate was lower in athlete groups compared to controls (Table 1).

Interventricular septal thickness, posterior wall thickness, and left ventricular internal diameter were higher in water polo athletes compared to both fitness athletes and controls (Table 2). Relative wall thickness did not differ between groups. E and A waves of mitral inflow, E/A ratio, deceleration time, and mitral annular septal and lateral diastolic velocities, as well as E/e′ ratio, were comparable among the groups referring to similar diastolic function. LA volume and LA volume index were higher in water polo athletes, while being comparable between fitness athletes and controls. Conventional linear measurements of RV geometry and function showed no significant difference among groups. TAPSE and tricuspid annular systolic and diastolic velocities were also similar. RA volume and RA volume index were higher only in water polo athletes compared to the sedentary volunteer group (Table 2).

**Figure 1:**
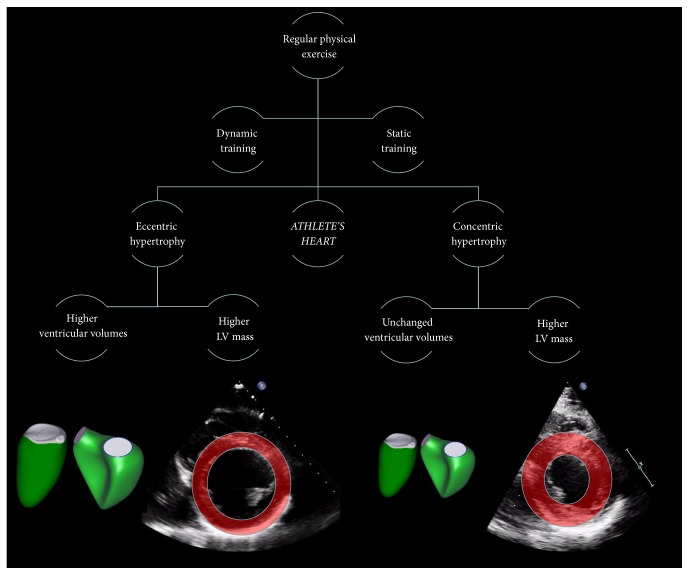
Schematic representation of our results. More dynamic exercise training induced eccentric hypertrophy in female water polo athletes, with higher left and right ventricular volumes (3D echocardiography derived models, respectively) and also higher left ventricular (LV) mass (parasternal short axis view of the left ventricle showing dilation and higher mass). On the other hand, mainly static exercise training induced concentric hypertrophy in female fitness athletes, with unchanged ventricular volumes but higher left ventricular mass (parasternal short axis view of the left ventricle showing higher mass without dilation).

Fitness athletes presented similar left ventricular end-diastolic, end-systolic, and stroke volumes compared to healthy, sedentary volunteers (Table 3). Water polo athletes, however, had higher LV end-diastolic and end-systolic volumes even after indexing to BSA. Correspondingly, LV EF was similar in fitness athletes compared to controls, while it was lower in water polo athletes. LV stroke volume and stroke volume index did not differ between groups. LV mass and mass index were significantly higher in the athlete groups; the hypertrophy, however, was even more prominent in water polo athletes (Figure 1). Referring to the geometrical changes, global longitudinal and circumferential strains were lower in water polo athletes. Systolic deformation parameters were similar between fitness athletes and controls. Similarly, RV end-diastolic, end-systolic, and stroke volumes were all similar in fitness athletes and controls, while they were higher in water polo athletes. RV EF showed no difference between the groups and neither did FAC and free wall longitudinal strain, referring to similar systolic function of the RV (Table 3).

In fitness athletes, FFMI correlated with RV EDV (*r* = 0.607, *p* < 0.05), RV SV (*r* = 0.647, *p* < 0.05), and RV length (*r* = 0.575, *p* < 0.05); in water polo athletes weekly training time correlated with LV mass (*r* = 0.527, *p* < 0.05) and the number of years spent with professional sport activity with LVMi (*r* = 0.567, *p* < 0.05).

## 4. Discussion

In our study, we aimed at comparing two different sport disciplines in the context of female athlete's heart using 3D echocardiography. In IFBB Bikini Fitness athletes, a mild, concentric-type of LV hypertrophy is present, while in water polo athletes eccentric LV hypertrophy develops (Figure 1). To the best of our knowledge, our study is the first to characterize athlete's heart of Bikini Fitness competitors and also to suggest the applicability of Morganroth's hypothesis in women.

Athlete's heart is first and foremost characterized by a physiological increase in LV mass [1, 3]. Morganroth's classical hypothesis suggests that sports with mainly endurance exercise nature result in eccentric LV hypertrophy, while power sports induce concentric hypertrophy [6]. However, the spectrum of athlete's heart is very broad and substantive investigation of the adaptation induced by mostly endurance or power training is difficult, especially among women [4, 12]. Therefore, we selected our study population to address this issue. Water polo is a good example of mixed exercise training with more dynamic components and a very high training load (>20 hours/week) with international antidoping protocols in effect. The goal of IFBB Bikini Fitness athletes, however, is completely different: to sculpt a muscular, defined and toned, healthy looking physique with a reasonable amount of muscle mass [5]. Training regime of these fitness athletes comprises mainly relatively short duration but markedly high intensity static exercises, with dynamic components and overall training time being limited to avoid unwanted muscle mass loss. The use of performance and muscle enhancing doping is also strictly audited by the IFBB and is also counterproductive in this category. To date, no study has investigated this increasingly popular sport. We have found that female athlete's heart of fitness competitors is characterized by mild, concentric-type LV hypertrophy compared to the significantly higher amount of LV mass and eccentric hypertrophy presented by female water polo athletes. LV and RV systolic or diastolic function was found to be unchanged in fitness athletes compared to healthy, sedentary volunteers.

Nevertheless, selection of imaging modality to delineate even subtle alterations in cardiac morphology and function is of pivotal importance. 3D echocardiography was shown to have better correlation with gold standard cardiac MRI compared to conventional M-mode and 2D echocardiographic measurements [13–15]. The technical setup is essential in this regard since, for example, LV wall thickness values did not show difference between fitness athletes and controls in our study. 3D echocardiography, however, was able to show LV hypertrophy of fitness athletes. The same applies to LV and RV volumetric measurements. For example, simple linear RV parameters failed to indicate difference even between water polo athletes and healthy controls; however, 3D echocardiography showed a marked RV dilation in water polo athletes which corresponds to previous literature and their nature of exercise [16]. This highlights the usefulness of 3D echocardiography in measuring chamber volumes and LV mass in the athlete's heart. In water polo athletes furthermore, we were able to show the correlation between the time of training and gain of LV mass.

Exercise-induced dilation of the ventricles often leads to low-normal resting values regarding functional parameters [17, 18]. In our cohort, water polo athletes had lower LV EF along with decreased longitudinal and circumferential systolic deformation compared to both healthy volunteers and fitness athletes. The increased LV contractility of athlete's heart is a well-known phenomenon; however, resting echocardiographic parameters (including strains) are not always able to explore this in every scenario, resulting in conflicting results in the literature [19]. Lo Iudice and colleagues found, however, increased LV longitudinal, circumferential, and area strains in male endurance athletes, with sinus bradycardia, increased LV mass, and afterload emerging as major determinants of these changes [20]. Notably, Monte et al. found significantly lower strain values compared to sedentary controls in a set of predominantly male, strength-trained athletes [21]. Despite the lower values of systolic function parameters in our water polo athletes, LV stroke volume and stroke volume index were similar among groups. Regarding the RV, EF, and free wall longitudinal strain remained comparable in both athlete groups to sedentary volunteers, resulting in a higher SV and SVi in water polo athletes (along with the RV dilation). However, there are also reports on RV supernormal longitudinal function in top level male rowers [22], which may suggest a shift in the relative contribution of different RV wall motion components in athletes [23]. It has been recently shown in female athletes that exercise-induced cardiac remodeling appears in a balanced manner at both the interventricular and atrioventricular levels, yet correlating with the intensity of dynamic exercise [24]. Our results are in line with these observations.

Diastolic function of athlete's heart may be also an important feature, as even resting measures may be able to indicate the supernormal function of athletes and moreover to differentiate between physiological and pathological hypertrophies [25]. In our current study, the three investigated groups were similar in terms of all diastolic function parameters. Similar to the dilation of the ventricles, left and right atria were also significantly larger in water polo athletes, while they were comparable between fitness athletes and controls. Biatrial dilation of endurance athletes is also an established phenomenon along with known gender differences in it [26].

We have also assessed body composition to characterize muscle gain of fitness athletes. Fat-free mass index (FFMI) is a popular parameter among bodybuilders because it reflects better muscle mass gain than BMI [27, 28]. Moreover, high values may also refer to anabolic steroid abuse and could be used for screening purposes [27]. Our results of athletes are typical for healthy, nonuser athletes and the values of control subjects also correspond well to previous normative studies [28]. Although the water polo athletes had higher height and weight, fitness athletes presented with even higher FFMI showing the remarkable muscle mass gain related to this sport discipline. Interestingly, we have found significant correlations between FFMI and RV, but not LV remodeling, which may suggest potential effects of static exercise training on RV morphology and function [29].

*Limitations. *Firstly, the low number of investigated athletes represents a limitation. However, our approach was to select two distinct populations from two different sport disciplines representing the highest level possible. Water polo athletes had a longer training history and more extensive weekly training sessions compared to fitness athletes and their exercise nature is mixed rather than clearly dynamic. These factors may bias our comparison. Only resting echocardiographic measurements were performed in our current study. Further investigations are warranted to characterize cardiac function of female fitness athletes during exercise. Our study has a cross-sectional design, while the temporal changes in LV and RV volumes and mass, such as throughout a training season, or dynamics of deconditioning remain unknown. Although 3D echocardiographic solutions are commercially available, LA and RA volumes were calculated by a 2D method in our study. Despite the clear advantages of 3D echocardiography, still cardiac MRI is the gold standard method for the quantification of LV and RV volumes and mass.

## 5. Conclusions

To the best of our knowledge, this is the first study to characterize female athlete's heart of IFBB Bikini Fitness competitors. The predominantly static exercise regime induced a mild, concentric-type hypertrophy, while in water polo athletes higher ventricular volumes and eccentric LV hypertrophy developed. Fitness athletes presented unchanged LV and RV systolic and diastolic function compared to sedentary volunteers. These findings highlight the applicability of Morganroth's classical hypothesis in the context of female athlete's heart.

## Figures and Tables

**Table 1:** Basic demographic and anthropometric characteristics of the study groups.

	Fitness athletes	Water polo athletes	Healthy controls	ANOVA *p*
*n*	15	15	15	
Age (years)	24** ± **3	24** ± **4	23** ± **2	0.357
Height (m)	1.63** ± **0.05^*∗*^	1.75** ± **0.06^#§^	1.66** ± **0.06^*∗*^	<0.0001
Weight (kg)	57.0** ± **7.1^*∗*^	65.7** ± **5.4^#§^	56.6** ± **8.3^*∗*^	<0.001
BMI (kg/m^2^)	21.3** ± **1.9	21.4** ± **1.2	20.5** ± **2.8	0.399
BSA (m^2^)	1.6** ± **0.1^*∗*^	1.8** ± **0.1^#§^	1.6** ± **0.1^*∗*^	<0.0001
FFMI (kg/m^2^)	18.2** ± **1.8^*∗*§^	17.1** ± **1.1^#§^	14.9** ± **1.4^*∗*#^	0.001
Systolic blood pressure (mmHg)	115** ± **14^*∗*^	135** ± **12^#§^	122** ± **10^*∗*^	<0.0001
Diastolic blood pressure (mmHg)	74** ± **9	76** ± **6	76** ± **2	0.684
Heart rate (/min)	63** ± **9^§^	69** ± **14^§^	82** ± **7^*∗*#^	0.003

BMI: body mass index; BSA: body surface area; FFMI: fat-free mass index. ^*∗*^Significant versus water polo athletes; ^#^significant versus fitness athletes; ^§^significant versus controls.

**Table 2:** Comparison of conventional echocardiographic measurements among the groups.

	Fitness athletes (*n* = 15)	Water polo athletes (*n* = 15)	Healthy controls (*n* = 15)	ANOVA *p*
IVSd (mm)	7.5** ± **0.9^*∗*^	9.5** ± **1.5^#§^	7.0** ± **0.9^*∗*^	<0.0001
LVPWd (mm)	7.2** ± **1.5^*∗*^	8.3** ± **0.8^#§^	6.6** ± **1.1^*∗*^	0.006
LVIDd (mm)	45.6** ± **4.6^*∗*^	49.0** ± **2.9^#§^	43.6** ± **3.3^*∗*^	0.005
RWT	0.32** ± **0.07	0.34** ± **0.04	0.31** ± **0.06	0.416
E wave (cm/s)	78.4** ± **21.4	78.9** ± **7.9	93.1** ± **16.4	0.115
A wave (cm/s)	54.1** ± **13.5	49.6** ± **14.9	58.8** ± **13.9	0.359
E/A ratio	1.64** ± **0.34	1.73** ± **0.57	1.67** ± **0.50	0.890
DCT (cm/s)	164.1** ± **49.5	190.1** ± **41.2	171.8** ± **25.9	0.261
mitral lateral annulus s′ (m/s)	0.10** ± **0.03	0.11** ± **0.01	0.12** ± **0.03	0.058
mitral lateral annulus e′ (m/s)	0.17** ± **0.04	0.18** ± **0.03	0.18** ± **0.06	0.686
mitral lateral annulus a′ (m/s)	0.08** ± **0.03	0.08** ± **0.02	0.08** ± **0.01	0.896
mitral medial annulus s′ (m/s)	0.09** ± **0.02	0.09** ± **0.01	0.10** ± **0.03	0.160
mitral medial annulus e′ (m/s)	0.13** ± **0.03	0.13** ± **0.01	0.15** ± **0.04	0.205
mitral medial annulus a′ (m/s)	0.08** ± **0.02	0.07** ± **0.01	0.08** ± **0.02	0.078
E/e′ average	4.76** ± **1.25	4.43** ± **0.85	5.90** ± **3.02	0.147
LA volume (ml)	30.1** ± **11.2^*∗*^	47.9** ± **10.6^#§^	28.6** ± **6.0^*∗*^	0.007
LA volume index (ml/m^2^)	17.4** ± **8.0^*∗*^	27.4** ± **7.2^#§^	18.1** ± **3.7^*∗*^	0.026
RVID base (mm)	38.1** ± **4.7	41.1** ± **5.7	36.2** ± **5.1	0.241
RVID mid (mm)	32.5** ± **3.9	36.1** ± **4.2	29.3** ± **4.2	0.076
RV length (mm)	81.6** ± **10.5	88.4** ± **12.1	76.2** ± **14.0	0.107
TAPSE (mm)	22.6** ± **6.2	24.9** ± **3.1	24.8** ± **3.8	0.362
tricuspid annulus s′ (m/s)	0.14** ± **0.03	0.12** ± **0.02	0.14** ± **0.03	0.054
tricuspid annulus e′ (m/s)	0.15** ± **0.02	0.15** ± **0.04	0.18** ± **0.04	0.099
tricuspid annulus a′ (m/s)	0.10** ± **0.02	0.08** ± **0.02	0.09** ± **0.02	0.162
RA volume (ml)	36.9** ± **11.9	46.2** ± **8.7^§^	28.7** ± **11.9^*∗*^	0.001
RA volume index (ml/m^2^)	21.5** ± **8.9	25.8** ± **7.6^§^	17.7** ± **6.4^*∗*^	0.011

IVSd: interventricular septal thickness in end-diastole; LVPWd: left ventricular posterior wall thickness in end-diastole; LVIDd: left ventricular internal diameter in end-diastole; RWT: relative wall thickness; DCT: deceleration time; LA: left atrium; RVID: right ventricular internal diameter; TAPSE: tricuspid annular plane systolic excursion; RA: right atrium. ^*∗*^Significant versus water polo athletes; ^#^significant versus fitness athletes; ^§^significant versus controls.

**Table 3:** Comparison of 3D echocardiographic measurements among the groups.

	Fitness athletes (*n* = 15)	Water polo athletes (*n* = 15)	Healthy controls (*n* = 15)	ANOVA *p*
LV EDV (ml)	121** ± **18^*∗*^	149** ± **15^#§^	115** ± **11^*∗*^	<0.0001
LV EDVi (ml/m^2^)	76** ± **13^*∗*^	84** ± **8^#§^	73** ± **8^*∗*^	0.045
LV ESV (ml)	45** ± **12^*∗*^	65** ± **10^#§^	40** ± **8^*∗*^	<0.0001
LV ESVi (ml/m^2^)	28** ± **8^*∗*^	36** ± **5^#§^	25** ± **4^*∗*^	0.002
LV EF (%)	63** ± **6^*∗*^	57** ± **5^#§^	65** ± **6^*∗*^	0.006
LV SV (ml)	76** ± **12	85** ± **12	75** ± **10	0.102
LV SVi (ml/m^2^)	48** ± **7	48** ± **7	48** ± **8	1.000
LVM (g)	125** ± **20^*∗*§^	163** ± **23^#§^	90** ± **15^*∗*#^	0.006
LVMi (g/m^2^)	78** ± **13^*∗*§^	91** ± **10^#§^	57** ± **10^*∗*#^	<0.0001
GLS (%)	−22.2** ± **3.2^*∗*^	−18.8** ± **1.6^#§^	−23.1** ± **1.7^*∗*^	<0.0001
GCS (%)	−30.6** ± **4.5	−27.2** ± **4.2^§^	−34.4** ± **4.9^*∗*^	0.006
RV EDV (ml)	99** ± **21^*∗*^	154** ± **26^#§^	88** ± **17^*∗*^	<0.0001
RV EDVi (ml/m^2^)	61** ± **12^*∗*^	86** ± **14^#§^	55** ± **9^*∗*^	<0.0001
RV ESV (ml)	40** ± **9^*∗*^	69** ± **16^#§^	35** ± **8^*∗*^	<0.0001
RV ESVi (ml/m^2^)	25** ± **6^*∗*^	39** ± **9^#§^	22** ± **4^*∗*^	<0.0001
RV EF (%)	59** ± **6	56** ± **5	60** ± **4	0.129
RV SV (ml)	58** ± **15^*∗*^	85** ± **12^#§^	52** ± **10^*∗*^	<0.0001
RV SVi (ml/m^2^)	36** ± **9^*∗*^	48** ± **7^#§^	33** ± **6^*∗*^	<0.0001
FAC (%)	50** ± **7	49** ± **7	54** ± **4	0.312
Free wall longitudinal strain (%)	−31.1** ± **5.0	−33.5** ± **4.4	−30.7** ± **4.7	0.325

LV: left ventricular; EDVi: end-diastolic volume index; ESVi: end-systolic volume index; EF: ejection fraction; SVi: stroke volume index; LVMi: left ventricular mass index; GLS: global longitudinal strain; GCS: global circumferential strain; RV: right ventricular; FAC: fractional area change. ^*∗*^Significant versus water polo athletes; ^#^significant versus fitness athletes; ^§^significant versus controls.

## Data Availability

Datasets are available from corresponding author upon request.
